# Emotional processing in Parkinson’s disease and schizophrenia: evidence for response bias deficits in PD

**DOI:** 10.3389/fpsyg.2015.01417

**Published:** 2015-09-24

**Authors:** Ilona P. Laskowska, Ludwika Gawryś, Szymon Łęski, Dariusz Koziorowski

**Affiliations:** ^1^Music Performance and Brain Laboratory, Department of Cognitive Psychology, University of Finance and Management, WarsawPoland; ^2^2nd Department of Psychiatry, Institute of Psychiatry and Neurology, WarsawPoland; ^3^Laboratory of Neuroinformatics, Department of Neurophysiology, Nencki Institute of Experimental Biology, WarsawPoland; ^4^Department of Neurology, Faculty of Health Science, Medical University of Warsaw, WarsawPoland

**Keywords:** facial emotion recognition, mild cognitive impairment, Parkinson’s disease, response bias, schizophrenia, signal detection theory

## Abstract

Deficits in facial emotion recognition in Parkinson’s disease (PD) patients has been well documented. Nevertheless, it is still not clear whether facial emotion recognition deficits are secondary to other cognitive impairments. The aim of this study was to answer the question of whether deficits in facial emotion recognition in PD result from impaired sensory processes, or from impaired decision processes. To address this question, we tested the ability to recognize a mixture of basic and complex emotions in 38 non-demented PD patients and 38 healthy controls matched on demographic characteristics. By using a task with an increased level of ambiguity, in conjunction with the signal detection theory, we were able to differentiate between sensitivity and response bias in facial emotion recognition. Sensitivity and response bias for facial emotion recognition were calculated using a *d-prime* value and a *c* index respectively. Our study is the first to employ the EIS-F scale for assessing facial emotion recognition among PD patients; to test its validity as an assessment tool, a group comprising schizophrenia patients and healthy controls were also tested. Patients with PD recognized emotions with less accuracy than healthy individuals (*d-prime*) and used a more liberal response criterion (*c* index). By contrast, patients with schizophrenia merely showed diminished sensitivity (*d-prime*). Our results suggest that an impaired ability to recognize facial emotions in PD patients may result from both decreased sensitivity and a significantly more liberal response criteria, whereas facial emotion recognition in schizophrenia may stem from a generalized sensory impairment only.

## Introduction

In recent years, there has been increasing interest in the wide range of cognitive symptoms accompanying neurodegenerative disorders. This trend is reflected in the recommendations published in the DSM-5 (American Psychiatric Association, 2013), which state that neuropsychological assessment for such disorders should be expanded to include social cognition. This recommendation pertains to both mild and major neurocognitive disorders. Mild neurocognitive disorder (mNCD) in the domain of social cognition is defined as: “subtle changes in behavior or attitude, often described as a change in personality, such as less ability to recognize social cues or read facial expressions” (p. 595). For the assessment of patient competency in social cognition, the DSM-5 recommends evaluation in two domains: (1) those which measure the ability to recognize a variety of both positive and negative emotions, and (2) those which measure the ability to consider the mental state and experiences of others.

Parkinson’s disease (PD) is one such neurodegenerative disorder in which facial recognition impairment has frequently been identified (Dujardin et al., 2004; Ariatti et al., 2008; Baggio et al., 2012; Bediou et al., 2012). PD results from a loss of dopamine neurons in the pars compacta region of the substantia nigra and depletion of some of the neurons within the ventral tegmental area (Braak et al., 2003; Drui et al., 2014). These degenerations affect both nigrostriatal and mesocorticolimbic systems and seem to be associated with facial emotion recognition ability in PD (Péron et al., 2012). The most commonly occurring impairments are seen in the ability to recognize basic negative emotions: fear, sadness, anger, and disgust (for review, see: Gray and Tickle-Degnen, 2010). Inability to recognize anger and disgust has been shown to be directly related to dopamine depletion (Sprengelmeyer et al., 2003; Lawrence et al., 2007).

There is still much debate as to whether emotion recognition impairment in PD is restricted to negative emotions (Suzuki et al., 2006; Gray and Tickle-Degnen, 2010). One recently published study (Buxton et al., 2013) found that PD patients did exhibit deficits in the ability to recognize happiness. In that study, six basic emotions were presented at three levels of intensity: low, medium and high. Results show that the PD group’s ability to identify happiness was affected when the intensity level of the emotion was decreased to medium or low. A similar pattern is not observed with negative emotions. Buxton’s results indicate that impairment in facial emotion recognition among PD patients is (1) not restricted to negative emotions, and (2) dependent upon the intensity of the stimulus. Impairment in the recognition of complex emotions (so-called “social emotions”) has also been documented. One study found that the ability to recognize arrogance was reduced among PD patients following temporary withdrawal from dopamine replacement therapy (Martins et al., 2008). Such findings raise the question as to whether additional cognitive processes are involved when faced with ambiguous stimuli, such as basic emotions with reduced intensity or more complex emotions.

Impaired performance on various cognitive tasks is well described in PD patients (Verbaan et al., 2007; Muslimovic et al., 2009). Executive dysfunctions, which are extremely common (Owen, 2004; Kudlicka et al., 2011; Ravizza et al., 2012) even in the early stages of the disease (Levin and Katzen, 2005), include difficulties with decision-making, categorization, executive attention, and working memory.

A small number of studies have investigated the possibility that emotion recognition impairment in PD is secondary to executive dysfunction. However, due to the paucity of data, no consistent conclusion could be drawn (Gray and Tickle-Degnen, 2010). What is more, most of these studies (e.g., Breitenstein et al., 1998; Pell and Leonard, 2005; Clark et al., 2008; Herrera et al., 2011) used different neuropsychological measures (e.g., verbal fluency tasks, Trail Making Test (TMT), Wisconsin Card Sorting Test, Visual Search) to examine different aspects of executive function. However, the failure of these studies to find a link between emotion recognition impairment and executive dysfunction does not negate the possibility that such a link exists. To investigate the processes underlying facial emotion recognition, it may be useful to employ a method which already incorporates some aspects of executive function (e.g., decision-making) and explores the range of emotions encountered in everyday life. Such a method would therefore include tasks which present a degree of uncertainty and would make demands upon an individual’s decision-making processes. According to Krantz (1969), decision-making comprises sensory processes and cognitive decision processes. Signal detection theory (SDT) is a useful tool for analyzing both these measures of performance (Stanislaw and Todorov, 1999; Macmillan and Creelman, 2004). Decision-making strategy is measured in terms of response bias, whereas the accuracy of stimulus detection is expressed in terms of sensitivity. The distinction between sensitivity and response bias appears to be particularly significant when tasks include a higher level of difficulty and when response strategies play an important role due to greater ambiguity of stimuli.

To date, the SDT has only been applied in a few studies pertaining to facial emotion recognition (e.g., Tsoi et al., 2008; Pixton, 2011; Huang et al., 2013). To the best of our knowledge, it has been used in only one study involving PD patients (Narme et al., 2011). However, Narme et al. (2011) were primarily interested in specific visuospatial deficits than in executive function deficits. They hypothesized that facial emotion recognition impairment in PD may result from configural processing deficits. Their study included the following tasks: (1) a facial emotion recognition task; (2) an upside-down facial emotion recognition task, and (3) a facial configuration detection task. Their results showed that configural performance was positively correlated with emotion recognition of negative emotions. It has been suggested that impaired recognition of emotion from facial cues could be related, at least partially, to configural processing alteration, especially for vertical, second-order information. However, these results are in contrast to previous studies examining visuospatial deficits among PD patients (for review, see: Gray and Tickle-Degnen, 2010), which suggests that facial emotion recognition deficits in PD are independent from general deficits in face processing. These discrepancies could be accounted for by task differences, since major studies assessed visuospatial deficits with the Benton Facial Recognition Task, which was not designed specifically to serve as a measure of configural processing. It is worth noting that decision-making deficits, categorization impairments and decreased working memory in PD were not controlled in the study by Narme et al. (2011). However, the authors of that study do not disregard the need to clarify the role of attention and executive functions in more complex experimental tasks which demand specific cognitive activity in future studies (Narme et al., 2011).

In our study we choose not to focus on emotion-specific recognition deficits, opting instead to assess emotion recognition deficits using a wider range of emotions. We tested the ability of PD patients to recognize a mixture of basic and complex emotions. To do this, we employed the Emotional Intelligence Scale – Faces (EIS-F), which complies with the recommendations of the DSM-5. As far as we know, our study is the first to assess the usefulness of EIS-F for research into cognitive impairment in patients with neurological and psychiatric disorders.

The aims of our study were as follows: (1) to assess facial emotion recognition among patients with PD, using a task which is more ecologically valid than those which merely assess basic emotions. This will give us a more accurate picture of the ability of these patients to recognize facial emotions in a natural environment; (2) to answer the question of whether deficits in facial emotion recognition in PD result from impaired sensory processes (i.e., decreased sensitivity in stimulus detection), or from a decision-making impairment (measured as response bias). By using a task with a greater level of ambiguity in conjunction with the SDT, we should be able to differentiate sensory process deficits from decision-making deficits; (3) to assess the diagnostic accuracy of EIS-F and to ascertain its usefulness as an assessment tool for mNCD.

In order to check the validity of the EIS-F for assessing facial emotion recognition among PD patients, a control group comprising schizophrenia patients was also tested. As with PD patients, a number of studies have shown that patients with schizophrenia exhibit impaired facial emotion recognition, compared with healthy controls (Feinberg et al., 1986; Archer et al., 1992; Salem et al., 1996; Addington and Addington, 1998; Kohler et al., 2003). It has been observed that impairment of emotion recognition in both PD patients (e.g., Dujardin et al., 2004; Lawrence et al., 2007; Clark et al., 2008) and SCH patients (e.g., Bediou et al., 2005) results from a disturbed dopaminergic system. There is some evidence that there is an inverted U shaped relationship between emotion recognition ability and dopamine level (Delaveau et al., 2005). In one study (Delaveau et al., 2005), healthy individuals were given levodopa and this had an effect on amygdala activation during the performance of the facial emotion recognition task. We can therefore expect that diminished dopaminergic innervation of the amygdala in PD, and dopamine overstimulation in SCH, will have a negative impact on emotion recognition ability. Furthermore, it is worth noting that people with schizophrenia, in contrast to PD patients, may show a more general deficit in face perception (for review, see: Bortolon et al., 2015). Performance in the BVRT and similar tasks is usually impaired in schizophrenia patients, compared to healthy controls. According to Bortolon et al. (2015) this difference was not seen in only four studies, while schizophrenia patients displayed impaired performance in over a dozen studies. In the case of PD patients, the exact opposite pattern of results is found: Gray and Tickle-Degnen (2010) found that in 15 studies examining BVRT performance, there was no difference between PD patients and healthy controls, and only four showed impaired performance in the PD group. The results of meta-analysis performed by the authors of those studies suggest that the existence of facial emotion recognition impairment in PD cannot be explained in terms of a general visuospatial deficit. We therefore included the SCH group as an additional control group that would be expected to exhibit decreased discriminability of facial emotions. We expected robust visual face processing deficits to have a significant effect on performance in the EIS-F, given that the task requires discrimination of subtle facial features. We wanted to find out if decreased sensitivity to facial emotions is accompanied by changes in response strategy. As already mentioned, the SDT is rarely used in emotion recognition studies and the link between response strategy and ability to discriminate emotions has not been determined. Our goal was to study both of these factors and, in including the SCH group, we would be able to determine response strategy changes in the context of sensitivity changes.

## Materials and Methods

### Participants

Thirty-eight non-demented patients with Parkinson’s disease (14 females) and 38 healthy controls matched for sex, age, and education took part in the study. PD patients were recruited via the Parkinson’s Disease Association in Bydgoszcz and the Parkinson’s Disease Foundation in Warsaw. All patients met the criteria for Idiopathic Parkinson’s disease with mild or medium intensity motor symptoms, as per the Hoehn and Yahr Scale (mean = 2.34, *SD* = 1). All patients were treated with levodopa or a dopamine agonist medication, and were tested soon after medication was administered (i.e., during their “on” state). PD patients with a Mini-Mental State Examination (MMSE) score below 24 were excluded from the test group.

In addition to the above, a group of 26 patients with schizophrenia (nine females) were compared to 26 healthy controls matched for sex, age, and education. This group of patients was recruited from schizophrenia foundations in Bydgoszcz, Inowrocław, Sicienko, and Toruń. The schizophrenia patients were diagnosed in accordance with the Diagnostic and Statistical Manual of Mental Disorders-IV. Patients were being treated with antipsychotic medication at the time of testing. Schizophrenia patients with an MMSE score below 24 were excluded from the test group.

All participants were native speakers of Polish. Healthy controls were recruited from the general population. Measures of cognitive functions (MMSE) and depression (BDI) were administered prior to testing. **Table 1** shows the demographic and clinical characteristics of Parkinson’s disease and schizophrenia patients and their respective control groups. Informed written consent was obtained from all subjects prior to testing and the study was approved by the local ethics committee.

**Table 1:** Demographic, clinical and cognitive characteristics of the sample.

	PD		HC_PD_			
Variable	*M*	*SD*	*M*	*SD*	*t/U#*	*p*
Age	61.42	8.52	60.24	9.34	0.58	0.57
Gender M/F	24/14		24/14			–
Education (years)	12.76	3.43	13.18	3.42	678.5#	0.32
Disease duration	8.63	5.09				
BDI	11.84	7.43	6.13	6.40	344.5#	<0.001
MMSE	28.47	1.57	29.47	0.91	403.5#	<0.001
Str WR	33.79	14.64	35.00	12.76	534.0#	0.33
Str CW	89.71	37.55	70.50	20.62	318.0#	<0.001
TMT A	56.24	35.44	39.53	9.84	363.0#	<0.01
TMT B	137.39	99.47	72.83	45.55	267.5#	<0.001
TMT B-A	81.16	76.30	31.87	43.84	234.5#	<0.001
BVRT corrects	5.37	2.20	5.77	1.77	524.0#	0.28
BVRT errors	7.84	4.19	6.13	3.13	1.86	0.07
RAVLT 1-5	39.13	11.83	41.23	10.69	540.5#	0.36
RAVLT LTM	7.89	3.09	7.67	2.92	0.30	0.76

	**SCH**		**HC**_**SCH**_			
	***M***	***SD***	***M***	***SD***	***t/U#***	***p***

Age	37.46	10.90	38.04	13.66	337.0#	0.50
Gender M/F	17/9	–	17/9	–	–	–
Education (years)	12.83	3.48	13.33	2.69	277.0#	0.13
Disease duration	17.69	9.17				
BDI	11.17	11.94	8.23	8.64	282.0#	0.28
MMSE	28.42	1.81				

*PD, Parkinson’s disease; HC, healthy controls; M, mean; SD, standard deviation; t/U, t statistic or U statistic (for non-normally distributed samples); BDI, Beck Depression Inventory; MMSE, Mini-Mental State Examination; Str WR, CW, Stroop Color-Word Test (Word Card, Color-Word Card); TMT A, B, B-A, Trail Making Test part A and B, and difference; BVRT corrects, errors, Benton Visual Retention Test, number of correct answers, and number of errors; RAVLT 1-5, Rey Auditory Verbal Learning Test sum of trials 1-5, LTM, long term memory score; SCH, Schizophrenia patients.*

### Neuropsychological Assessment

In order to assess the accuracy of the EIS-F and its diagnostic utility for PD patients, we examined the relationship between emotional processing and cognitive functions. Each patient with PD and each control subject were given a set of neuropsychological tests. The assessment was performed by a neuropsychologist. To assess visual attention, psychomotor speed and alternating attention, we employed the TMT, which is a widely used tool for testing executive functions. TMT comprises parts A (number sequencing) and B (number-letter switching). We also calculated the TMT B-A index to remove the speed component. To examine selective attention and inhibition control, we used the Stroop Test (STR), which comprises a word-reading index (WR) and color-word naming index (CW), and is designed to assess cognitive speed and executive function. To test visual and verbal memory, we used the Benton Visual Retention Test (BVRT), which is designed to assess short-term visual memory, and the Rey Auditory Verbal Learning Test (RAVLT), which is widely used to assess episodic verbal memory. We used RAVLT trials 1–5 as a measure of verbal learning, and RAVLT ltm as an indicator of ability to retrieve information after a 20-min interval.

Mild cognitive impairment (MCI) was defined by neuropsychological testing as impaired performance (i.e., 2 SD below the mean score for the age- and education-matched control group) in two neuropsychological tests (see: Litvan et al., 2012). Subjective complaints of cognitive problems (or lack thereof) were not treated as a factor in the selection process.

### Facial Emotion Recognition Task

**FIGURE 1:**
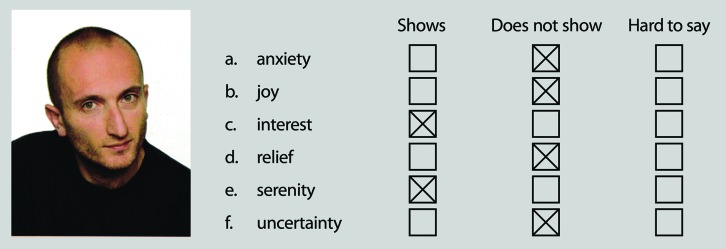
**Example photograph from Emotional Intelligence Scale – Faces (EIS-F) and correctly filled answer sheet (names of the emotions translated to English from the Polish original).** The photograph shown comes from the test instructions and does not feature in the actual test. Reprinted with permission.

Our study utilized the (EIS-F (Matczak et al., 2005). The test comprises 18 photographs, nine featuring male faces and nine featuring female faces. For each group of nine, four photographs depict positive emotions and five depict negative emotions. Accompanying each photograph is a list of six possible emotions (see **Figure 1**). The subject must determine which of the six emotions are shown in each photograph, and which are not, by choosing one of three possible responses: “shown,” “not shown,” and “hard to say.” Before the test commences, the subject is instructed that the “hard to say” response should only be given as a last resort. There are 108 items in total (i.e., 18 photographs × 6 names of emotions). The number of emotions expressed in each photograph is from one to four. Perfect score in the test requires the identification of 45 “shown” emotions and correct rejection of 63 “not shown” emotions.

The emotions depicted in the photographs include both basic emotions (positive: joy, surprise; negative: sadness, anxiety, anger, disgust), and complex emotions (positive: tenderness, self-contentment, pride, satisfaction, admiration, hope, coquetry, composure, self-confidence, curiosity, expectation, interest, astonishment; negative: unpleasant surprise, confusion, aversion, distrust, resignation, regret, disappointment, insecurity, disregard, feeling of superiority, indignation, envy, hate, contempt, unease, jealousy, disbelief).

### Data Analysis

In EIS-F, each decision as to whether a given emotion is present (or not) in a photograph is considered to be a separate answer in the test. The number of correct responses in the test is: “shown” = 45, “not shown” = 63. The authors of the test have proposed only one performance indicator, i.e., the total number of correct responses, be they “shown” or “not shown.” However, the summary result can be broken down into six possible responses: correct positive answer (“shown”), correct negative answer (“not shown”), incorrect positive answer (“shown”), incorrect negative answer (“not shown”), a response of “hard to say” when the correct response should have been “shown,” a response of “hard to say” when the correct response should have been “not shown.”

In our study, we analyzed the EIS-F results using the SDT (Macmillan, 2002). The SDT predicts that, for tasks which require a yes/no answer, performance is dependent upon the accuracy with which the subject discriminates between a known process (the signal) and chance (the noise). Moreover, the SDT takes into consideration the response strategy employed by the subject: where the subject experiences uncertainty, he may give a positive response (liberal strategy) or a negative response (conservative strategy). The EIS-F presents subjects with this choice, in that they must choose whether a given photograph depicts or does not depict the emotion in the accompanying list. In accordance with the SDT classification, correct positive responses (“shown”) are known as “hits.” Correct negative responses (“not shown”) are “correct rejections.” Incorrect positive responses (“shown”) are called “false alarms.” Incorrect negative responses (“not shown”) are known as “misses.” In the case of EIS-F, responses of “hard to say” prove problematic because the SDT does not take this option into consideration. However, since the “hard to say” responses are classed as erroneous in the EIS-F, such responses have also been classed as incorrect in our analysis. For this reason, a response of “hard to say” in cases where the correct response should have been “shown” are classed as misses. A response of “hard to say” when the correct response should have been “not shown” is classed as a false alarm.

In order to measure performance in a given task, the SDT uses the sensitivity index *d’* (Macmillan, 2002), which calculates the difference between hits and false alarms. The higher the value of the *d’*, the more accurate the distinction between signal and noise.

The second index used by the SDT is the response bias index c. Positive c index values indicate a conservative response strategy. In cases of uncertainty, the subject is more likely to give a negative response (expressed in EIS-F as a “not shown” response). A negative *c* index indicates a liberal response strategy, whereby the subject gives a positive response in cases of uncertainty (expressed in EIS-F as a “shown” response). We chose the *c* index, instead of the common β index, because it is not affected by changes in the *d’* (Ingham, 1970; Macmillan, 1993).

### Statistical Analysis

Analyses were performed using custom scripts written in Python programming language with packages for scientific computing: SciPy (Oliphant, 2007), sdt_metrics, and pandas. Group differences in demographic, clinical and cognitive characteristics and facial emotion recognition variables were analyzed using independent two-tailed *t*-tests for normally distributed variables, the Mann–Whitney test for non-normally distributed variables. Correlations between neuropsychological, demographic, clinical factors and facial emotion recognition variables (*d’*, *c*, hit rate, false alarm rate), were analyzed using Pearson’s correlations. Receiver Operating Characteristic (ROC) curves were plotted for each group (patients/controls).

## Results

### PD Patients

There were no significant differences between PD patients and HCs regarding demographic variables (see **Table 1**). Depression scores were significantly higher in the PD group. Patients scored significantly lower on the MMSE than the HCs. Significant differences were observed in the executive functions measures (TMT, STR). There were no significant differences in memory measures (RAVLT, BVRT).

The hit rate was considerably higher in the PD group than in the HC group (**Table 2**), although this was accompanied by a higher rate of false alarm responses. Despite the higher rate of hits, the *d’* sensitivity index (which indicates the accuracy of recognition) showed no difference between the groups. For both groups, a large number of hits were accompanied by an equally large number of false alarms. Both groups employed a liberal response strategy (as indicated by a negative *c* index). At the same time, the response bias was significantly higher (i.e., larger deviation from zero) among PD patients, which shows that there is a greater tendency to give positive responses in this group.

**Table 2:** Emotion recognition: PD patients vs. healthy controls-specific indicators as per SDT.

	PD		HC_PD_			
	*M*	*SD*	*M*	*SD*	*t/U#*	*p*
Hit rate	0.75	(0.11)	0.68	(0.15)	2.29	<0.05
False alarms rate	0.56	(0.14)	0.40	(0.13)	229.5#	<0.001
*d-prime*	0.56	(0.39)	0.80	(0.44)	-2.49	<0.05
*c*	-0.45	(0.34)	-0.12	(0.34)	-4.19	<0.001

*d-prime, sensitivity index; *c*, response bias index.*

### Patients with Schizophrenia

The performance of patients with schizophrenia in the EIS-F, as expressed by hit rate, did not differ significantly from that of the healthy controls, although the schizophrenia patients gave a significantly higher number of false alarm responses (**Table 3**). This resulted in a considerably lower sensitivity index for schizophrenia patients compared with healthy controls. Both groups employed a liberal response strategy and showed no statistically significant differences in *c* index.

**Table 3:** Emotion recognition: schizophrenia vs. healthy controls-specific indicators as per SDT.

	SCH		HC_SCH_			
	*M*	*SD*	*M*	*SD*	*t/U*#	*p*
Hit rate	0.71	(0.16)	0.75	(0.12)	-1.23	0.23
False alarms rate	0.45	(0.17)	0.39	(0.12)	1.28	0.21
*d-prime*	0.76	(0.42)	1.03	(0.39)	-2.41	<0.05
*c*	-0.22	(0.45)	-0.23	(0.31)	-0.08	0.94

*d-prime, sensitivity index; *c*, response bias index.*

### Receiver Operating Characteristic

**FIGURE 2:**
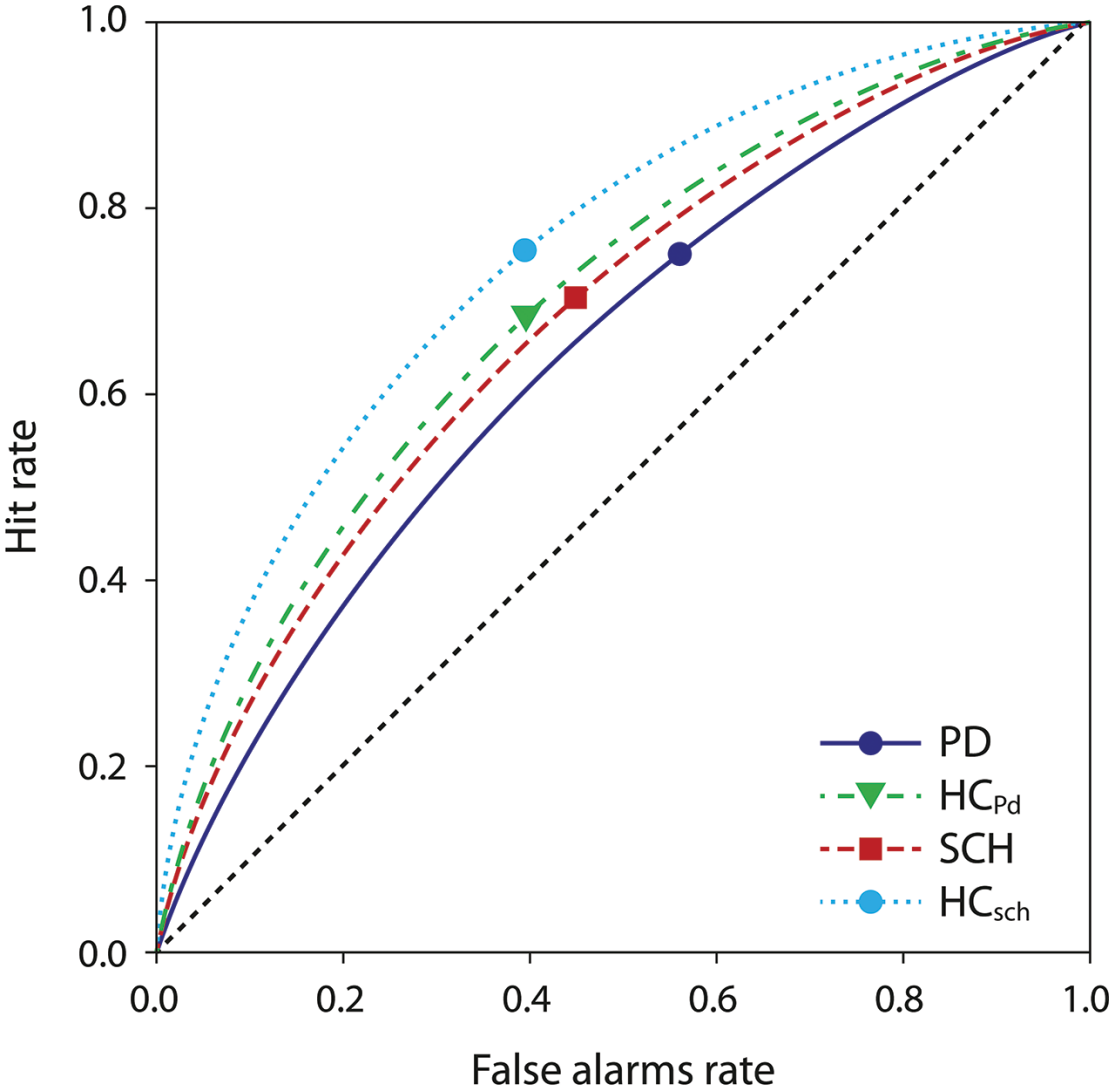
**Receiver operating characteristic (ROC) curves of the four groups included in the study.** Markers denote the mean false alarms rate and the mean hit rate of each group. Greater sensitivity (*d’*) corresponds to the ROC curve deflecting further from the diagonal; the position of the marker on the curve corresponds to response bias *c*. PD, PD patients, HC_PD_, control for PD group, SCH, schizophrenia patients, HC_SCH_, control for SCH group.

We plotted ROC curves defined by average hit and false alarm rates for each patient and control group, and calculated the *d’* index corresponding to these averages. As we can see in **Figure 2**, all ROC curves lie relatively close to the diagonal dotted line representing performance of random choice strategy. This suggests that the difficulty level of the task was relatively high. However, all groups perform above chance level. Note that younger HCs performed better than older HCs. This would indicate that the age of test subjects significantly affects facial emotion recognition ability.

The markers on the ROC curves represent average hit rate and false alarm rate within each group. Notably, the markers are positioned almost symmetrically for all groups (indicating little bias as measured by *c* index) except for the PD patient group, whose marker lies further to the right (higher hit and false alarm rates, larger deviation of *c* from zero).

### Correlations

In order to examine the relation between age and facial emotion recognition ability, a correlation analysis was performed for all healthy controls. We found a significant negative correlation between age and the *d’* (*r* = -0.42, *p* < 0.001). There was no significant correlation between age and *c* index (*r* = 0.13, *p* = 0.26). We also found no significant correlation between years of education and *d’* (*r* = 0.07, *p* = 0.67) or *c* index (*r* = -0.005, *p* = 0.98). Since the *c* index was lower among PD patients, we carried out a correlation analysis between facial emotion recognition variables and cognitive function measures. We found that there was a significant negative association between executive performance (TMT B-A) and response bias (*c* index) (*r* = -0.36, *p* < 0.05) and between TMT B-A and false alarm rate (*r* = 0.33, *p* < 0.05).

### Emotion Recognition in Relation to MCI

In order to provide MCI definition scores for PD, patients were categorized as pathological if their mean score was at least two standard deviations below that of the control group in two neuropsychological tests. Thus, 29 of the 38 PD patients were classified as cognitively intact and nine were classified as MCI. We compared emotion recognition performances of MCI and non-MCI PD patients and found no statistically significant differences.

## Discussion

Facial emotion recognition requires particular perception skills, such as an ability to discriminate facial features. However, whereas the most commonly used tests in research into emotion recognition consist of “yes/no”- type questions and answers, an equally important role is played by the cognitive decision process. In our study, we used the EIS-F to assess how both of these processes participate in facial emotion recognition in a group of individuals with PD, and in a group of individuals with schizophrenia. The SDT was used to measure perception and response bias. We found that: (1) patients with PD in comparison with age-matched healthy controls displayed sensory deficit in facial emotion recognition, as indicated by a decreased *d’* index value (2) individuals with PD employed a more liberal strategy than healthy individuals when it came to answering questions; (3) patients with schizophrenia showed less sensitivity in stimulus identification, compared with individuals from the age-matched healthy control group. Notably, decreased discriminability in the schizophrenia group was not accompanied by changes in response strategy, as indicated by the similar value of *c* index in the schizophrenia and healthy control groups. These findings indicate that facial emotion recognition ability can be sensitive to at least two potentially different process impairments, and the SDT may detect the impact of both sensory and executive deficits. Our findings are consistent with the belief that difficulties in facial emotion recognition in PD are not merely the result of a general deficit in face processing, but also the effect of executive control impairment (Péron et al., 2012). By contrast, facial emotion recognition difficulties in schizophrenia may stem from generalized perceptual impairment (Archer et al., 1992).

Patients with PD showed concurrent signs on the Stroop and TMT measures. It seems plausible that difficulty in processing emotions may stem from impaired executive functions. Indeed, we did find a correlation between the patients’ results on the facial emotion recognition task (*c* index, false alarms ratio) and TMT B-A measure. This finding suggests that the observation that prosodic emotion recognition in PD is partially dependent on deficits in executive functions (Gray and Tinkle-Degnen, 2010) and this also extends to facial emotion recognition. Moreover, we did not find any significant correlation between the *d’* index value and the results of executive function tests. Our results validate the specificity of *d’* and *c* measures, which are sensitive to impairments in two different processes.

With regards to sensory deficit in facial emotion recognition in PD, our findings were relatively similar to those of Narme et al. (2011). However, the overall discriminability of facial emotions in the study by Narme et al. (2011) was significantly higher than that observed in our study. The fact that we introduced stimuli of varying levels of difficulty (i.e., ambiguity and intensity) may explain this difference. This may also explain why both groups employed a highly conservative criterion (*c* = 0.4 in the PD group and *c* = 0.33 in healthy controls) in the study by Narme et al. (2011). Our use of complex facial emotions with lower intensity and higher ambiguity enabled us to detect changes in response strategy among PD patients, as expressed by a decreased *c* index value.

One could argue that the deficits in facial emotion recognition are an effect of general decline in cognitive functioning in PD. We excluded this possibility by comparing those patients with MCI with cognitively intact PD patients. We found that the difference in *d’* and *c* indices between the groups was not statistically significant. This finding concurs with the results of a study by Herrera et al. (2011), which revealed that emotion recognition impairment among PD patients was not related to the patients’ cognitive status (in both the PD MCI and PD non-MCI groups, approximately half of the patients displayed impaired facial emotion recognition).

For the HC groups, our results regarding the negative correlation of age and ability to discriminate facial emotions are broadly consistent with previous studies (Orgeta and Phillips, 2008; Ruffman et al., 2008); however, the stimuli in our study were a mixture of basic and complex emotions, and we did not analyze positive and negative emotions separately.

By using the SDT, we found that EIS-F is able to discriminate between patients with PD and HCs. In contrast to the majority of tests used to assess facial expression recognition, EIS-F measures a mixture of basic and complex emotions. Thus, the EIS-F test has the advantages of an ecological test, in which the ambiguity of an emotion does not merely result in a reduction in stimulus intensity. The test subject has to define more specific categories of meaning from the complex process of emotion classification. In other words, as well as having to decide whether a photograph depicts or does not depict a given emotion (e.g., a positive emotion), the subject must also define that emotion more precisely (e.g., pride, relief, flirtatiousness). For this reason, we can assume that the level of difficulty of the EIS-F will be high, an assumption confirmed by the results of the hit ratio, false alarm ratio, and *d’* values. It should be stressed that the SDT has, thus far, been seldom used in analyses of facial emotion recognition, and there are very few studies of the clinical population (Diehl-Schmid et al., 2007; Tsoi et al., 2008; Narme et al., 2011; Huang et al., 2013). That said, the existing literature tells us that the sensitivity index values obtained in our study were relatively low. The values ranged from 0.56 (mean for the PD group) to 1.03 (mean for the younger control group), and these values are similar to those obtained for healthy individuals in fast-paced (12.5–25 ms) basic emotion recognition tests (Pixton, 2011). In a study of schizophrenia patients (Tsoi et al., 2008), which also had a relatively short exposition time of 50 ms, the *d’* sensitivity index results were similar to those seen in our study. Another study of patients with schizophrenia, in which the faces shown to the subjects had been manipulated to exhibit different levels of intensity (Huang et al., 2013), the *d’* sensitivity index fell when the intensity of the stimulus was decreased. Even in groups of healthy individuals, the sensitivity index was lower than 1 when the intensity of a given emotion fell below 50%. The results of these studies suggest that the difficulty level of the EIS-F is indeed high, and is comparable to those tests which either limit the exposition time or considerably limit the intensity of the stimulus.

Future research should examine the sensitivity and accuracy of the EIS-F. One possible way of doing this is to check whether the results obtained in the EIS-F correlate with results obtained using other tests which measure facial emotion recognition. It would be extremely useful to do a comparison with more simply designed tests (Penn Emotional Facial Recognition – ER40) and considerably more difficult tests (e.g., tests where the level of intensity of presented emotions is manipulated).

Recognition of the social emotions used in the EIS-F requires not only efficient perception of a stimulus, but also efficient language competence. Our study was severely wanting in this regard, as we did not use any measurement of verbal comprehension (e.g., the relevant vocabulary subtest from the Wechsler Adult Intelligence Scale). However, this may be of more relevance to patients with schizophrenia than those with PD, given that language deficits are not typical among PD patients with cognitive dysfunctions (Goldman and Litvan, 2011). Still another limitation of this study is that there was no relevant neuropsychological measures to test whether the facial emotion recognition deficits in SCH could be due to a more specific cognitive impairment, even though we screened for patients’ global cognitive abilities using the Mini-Mental State Examination to exclude patients with a score below 24. Moreover, it has been noted that the stability of facial emotion recognition impairments over the course of schizophrenia may indicate an intermediate phenotype or an endophenotype of schizophrenia (Bediou et al., 2012), which suggests that facial emotion recognition impairments are not directly related to general cognitive function constraints. Also, since our study only examined PD patients and schizophrenia patients who were taking medication for their condition, the effect of non-pharmacological interventions on facial emotion recognition remains untested.

## Conclusion

Little research has been done into the process of “natural” social emotion recognition, since the majority of studies have used morphed faces in order to manipulate the intensity of emotions (e.g., by morphing two basic emotions in varying proportions). In doing so, variables may be strictly controlled (i.e., the proportion of an assessed emotion in the morphed stimulus). However, this does not fully reflect the natural emotions seen in everyday life. We do come across mixed emotions in our daily lives (e.g., anger mixed with sadness), but more often than not we are required to identify social emotions, such as mixtures of contempt and dislike, or admiration and pride. In our study we used the EIS-F, which has the advantages of an ecologically valid test measuring social emotion recognition. Our results suggest that: (1) PD significantly changes response bias and causes a slight decrease in sensitivity in the recognition of social emotions; (2) schizophrenia has very little effect on response bias, but is significantly connected with decreased sensitivity in the recognition of social emotions.

## Conflict of Interest Statement

The authors declare that the research was conducted in the absence of any commercial or financial relationships that could be construed as a potential conflict of interest.

## References

[B1] Addington J. Addington D. (1998). Facial affect recognition and information processing in schizophrenia and bipolar disorder. *Schizophr. Res.* 32 171–181. 10.1016/S0920-9964(98)00042-5 9720122

[B2] American Psychiatric Association. (2013). *Diagnostic and Statistical Manual of Mental Disorders: DSM-5^TM^*, 5th Edn. Arlington, VA: American Psychiatric Publishing, Inc.

[B3] Archer J. Hay D. C. Young A. W. (1992). Face processing in psychiatric conditions. *Br. J. Clin. Psychol.* 31 45–61. 10.1111/j.2044-8260.1992.tb00967 1559117

[B4] Ariatti A. Benuzzi F. Nichelli P. (2008). Recognition of emotions from visual and prosodic cues in Parkinson’s disease. *Neurol. Sci.* 29 219–227. 10.1007/s10072-008-0971-9 18810595

[B5] Baggio H. C. Segura B. Ibarretxe-Bilbao N. Valldeoriola F. Marti M. J. Compta Y. (2012). Structural correlates of facial emotion recognition deficits in Parkinson’s disease patients. *Neuropsychologia* 50 2121–2128. 10.1016/j.neuropsychologia.2012.05.020 22640663

[B6] Bediou B. Brunelin J. d’Amato T. Fecteau S. Saoud M. Hénaff M. A. (2012). A comparison of facial emotion processing in neurological and psychiatric conditions. *Front. Psychol.* 3:98 10.3389/fpsyg.2012.00098 PMC3318183 22493587

[B7] Bediou B. Franck N. Saoud M. Baudouin J. Y. Tiberghien G. Dalery J. (2005). Effects of emotion and identity on facial affect processing in schizophrenia. *Psychiatry Res.* 133 149–157. 10.1016/j.psychres.2004.08.008 15740991

[B8] Bortolon C. Capdevielle D. Raffard S. (2015). Face recognition in schizophrenia disorder: a comprehensive review of behavioral, neuroimaging and neurophysiological studies. *Neurosci. Biobehav. Rev.* 53 79–107. 10.1016/j.neubiorev.2015.03.006 25800172

[B9] Braak H. Del Tredici K. Rüb U. de Vos R. A. I. Jansen Steur E. N. H. Braak E. (2003). Staging of brain pathology related to sporadic Parkinson’s disease. *Neurobiol. Aging* 24 197–211. 10.1016/S0197-4580(02)00065-9 12498954

[B10] Breitenstein C. Daum I. Ackermann H. (1998). Emotional processing following cortical and subcortical brain damage: contribution of the fronto-striatal circuitry. *Behav. Neurol.* 11 29–42. 10.1155/1998/579029 11568400

[B11] Buxton S. L. MacDonald L. Tippett L. J. (2013). Impaired recognition of prosody and subtle emotional facial expressions in Parkinson’s disease. *Behav. Neurosci.* 127 193–203. 10.1037/a0032013 23565934

[B12] Clark U. S. Neargarder S. Cronin-Golomb A. (2008). Specific impairments in the recognition of emotional facial expressions in Parkinson’s disease. *Neuropsychologia* 46 2300–2309. 10.1016/j.neuropsychologia.2008.03.014 18485422 PMC2491661

[B13] Delaveau P. Salgado-Pineda P. Wicker B. Micallef-Roll J. Blin O. (2005). Effect of levodopa on healthy volunteers’ facial emotion perception: an FMRI study. *Clin. Neuropharmacol.* 28 255–261. 10.1097/01.wnf.0000186651.96351.2e 16340378

[B14] Diehl-Schmid J. Pohl C. Ruprecht C. Wagenpfeil S. Foerstl H. Kurz A. (2007). The Ekman 60 Faces Test as a diagnostic instrument in frontotemporal dementia. *Arch. Clin. Neuropsychol.* 22 459–464. 10.1016/j.acn.2007.01.024 17360152

[B15] Drui G. Carnicella S. Carcenac C. Favier M. Bertrand A. Boulet S. (2014). Loss of dopaminergic nigrostriatal neurons accounts for the motivational and affective deficits in Parkinson’s disease. *Mol. Psychiatry* 19 358–367. 10.1038/mp.2013.3 23399912 PMC5116056

[B16] Dujardin K. Blairy S. Defebvre L. Duhem S. Noël Y. Hess U. (2004). Deficits in decoding emotional facial expressions in Parkinson’s disease. *Neuropsychologia* 42 239–250. 10.1016/S0028-3932(03)00154-4 14644109

[B17] Feinberg T. E. Rifkin A. Schaffer C. Walker E. (1986). Facial discrimination and emotional recognition in schizophrenia and affective disorders. *Arch. Gen. Psychiatry* 43 276–279. 10.1001/archpsyc.1986.01800030094010 3954548

[B18] Goldman J. G. Litvan I. (2011). Mild cognitive impairment in Parkinson’s disease. *Minerva Med.* 102 441–459. 22193376 PMC3370887

[B19] Gray H. M. Tickle-Degnen L. (2010). A meta-analysis of performance on emotion recognition tasks in Parkinson’s disease. *Neuropsychology* 24 176–191. 10.1037/a0018104 20230112

[B20] Herrera E. Cuetos F. Rodríguez-Ferreiro J. (2011). Emotion recognition impairment in Parkinson’s disease patients without dementia. *J. Neurol. Sci.* 310 237–240. 10.1016/j.jns.2011.06.034 21752398

[B21] Huang C. L. Hsiao S. Hwu H. G. Howng S. L. (2013). Are there differential deficits in facial emotion recognition between paranoid and non-paranoid schizophrenia? A signal detection analysis. *Psychiatry Res.* 209 424–430. 10.1016/j.psychres.2013.03.026 23598059

[B22] Ingham J. G. (1970). Individual differences in signal detection. *Acta Psychol.* 34 39–50. 10.1016/0001-6918(70)90003-X 5454569

[B23] Kohler C. G. Turner T. H. Bilker W. B. Brensinger C. M. Siegel S. J. Kanes S. J. (2003). Facial emotion recognition in schizophrenia: intensity effects and error pattern. *Am. J. Psychiatry* 160 1768–1774. 10.1176/appi.ajp.160.10.1768 14514489

[B24] Krantz D. H. (1969). Threshold theories of signal detection. *Psychol. Rev.* 76 308–324. 10.1037/h0027238 5792074

[B25] Kudlicka A. Clare L. Hindle J. V. (2011). Executive functions in Parkinson’s disease: systematic review and meta-analysis. *Mov. Disord.* 26 2305–2315. 10.1002/mds.23868 21971697

[B26] Lawrence A. D. Goerendt I. K. Brooks D. J. (2007). Impaired recognition of facial expressions of anger in Parkinson’s disease patients acutely withdrawn from dopamine replacement therapy. *Neuropsychologia* 45 65–74. 10.1016/j.neuropsychologia.2006.04.016 16780901

[B27] Levin B. E. Katzen H. L. (2005). Early cognitive changes and nondementing behavioral abnormalities in Parkinson’s disease. *Adv. Neurol.* 96 84–94. 16383214

[B28] Litvan I. Goldman J. G. Tröster A. I. Schmand B. A. Weintraub D. Petersen R. C. (2012). Diagnostic criteria for mild cognitive impairment in Parkinson’s disease: movement Disorder Society Task Force guidelines. *Mov. Disord.* 27 349–356. 10.1002/mds.24893 22275317 PMC3641655

[B29] Macmillan N. A. (1993). “Signal detection theory as data analysis method and psychological decision model,” in *A Handbook for Data Analysis in the Behavioral Sciences: Methodological Issues*, eds Keren G. Lewis C. (Hillsdale, NJ: Erlbaum), 21–57.

[B30] Macmillan N. A. (2002). “Signal detection theory,” in *Stevens’ Handbook of Experimental Psychology: Methodology in Experimental Psychology*, 3rd Edn Vol. 4 eds Pasher H. Wixed J. (New York, NY: Wiley), 43–90.

[B31] Macmillan N. A. Creelman C. D. (2004). *Detection Theory: A User’s Guide*, 2nd Edn. Mahwah, NJ: Lawrence Erlbaum.

[B32] Martins A. Muresan A. Justo M. Simão C. (2008). Basic and social emotion recognition in patients with Parkinson disease. *J. Neurol. Sci.* 25 247–257.

[B33] Matczak A. Piekarska J. Studniarek E. (2005). *Skala Inteligencji Emocjonalnej – Twarze (SIE-T). Podręcznik.* Warszawa: Pracownia Testów Psychologicznych Polskiego Towarzystwa Psychologicznego.

[B34] Muslimovic D. Post B. Speelman J. D. De Haan R. J. Schmand B. (2009). Cognitive decline in Parkinson’s disease: a prospective longitudinal study. *J. Int. Neuropsychol. Soc.* 15 426–437. 10.1017/S1355617709090614 19402929

[B35] Narme P. Bonnet A.-M. Dubois B. Chaby L. (2011). Understanding facial emotion perception in Parkinson’s disease: the role of configural processing. *Neuropsychologia* 49 3295–3302. 10.1016/j.neuropsychologia.2011.08.002 21856319

[B36] Oliphant T. (2007). Python for scientific computing. *Comput. Sci. Eng.* 9 10–20. 10.1109/MCSE.2007.58

[B37] Orgeta V. Phillips L. H. (2008). Effects of age and emotional intensity on the recognition of facial emotion. *Exp. Aging Res.* 34 63–79. 10.1080/03610730701762047 18189168

[B38] Owen A. M. (2004). Cognitive dysfunction in Parkinson’s disease: the role of frontostriatal circuitry. *Neuroscientist* 10 525–537. 15534038 10.1177/1073858404266776

[B39] Pell M. D. Leonard C. L. (2005). Facial expression decoding in early Parkinson’s disease. *Brain Res. Cogn. Brain Res.* 23 327–340. 10.1016/j.cogbrainres.2004.11.004 15820640

[B40] Péron J. Dondaine T. Lejeune F. Grandjean D. Vérin M. (2012). Emotional processing in Parkinson’s disease: a systematic review. *Mov. Disord.* 27 186–199. 10.1002/mds.24025 22162004

[B41] Pixton T. S. (2011). Happy to see me, aren’t you, Sally? Signal detection analysis of emotion detection in briefly presented male and female faces. *Scand. J. Psychol.* 52 361–368. 10.1111/j.1467-9450.2011.00879.x 21447060

[B42] Ravizza S. M. Goudreau J. Delgado M. R. Ruiz S. (2012). Executive function abilities in Parkinson’s disease: contributions of the fronto-striatal pathways to action and feedback processing. *Cogn. Affect. Behav. Neurosci.* 12 193–206. 10.3758/s13415-011-0066-6 22006555

[B43] Ruffman T. Henry J. D. Livingstone V. Phillips L. H. (2008). A meta-analytic review of emotion recognition and aging: implications for neuropsychological models of aging. *Neurosci. Biobehav. Rev.* 32 863–881. 10.1016/j.neubiorev.2008.01.001 18276008

[B44] Salem J. E. Kring A. M. Kerr S. L. (1996). More evidence for generalized poor performance in facial emotion perception in schizophrenia. *J. Abnorm. Psychol.* 105 480–483. 10.1037/0021-843X.105.3.480 8772021

[B45] Sprengelmeyer R. Young A. W. Mahn K. Schroeder U. Woitalla D. Büttner T. (2003). Facial expression recognition in people with medicated and unmedicated Parkinson’s disease. *Neuropsychologia* 41 1047–1057. 10.1016/S0028-3932(02)00295-6 12667540

[B46] Stanislaw H. Todorov N. (1999). Calculation of signal detection theory measures. *Behav. Res. Methods Instrum. Comput.* 31 137–149. 10.3758/BF03207704 10495845

[B47] Suzuki A. Hoshino T. Shigemasu K. Kawamura M. (2006). Disgust-specific impairment of facial expression recognition in Parkinson’s disease. *Brain* 129 707–717. 10.1093/brain/awl011 16415306

[B48] Tsoi D. T. Lee K. H. Khokhar W. A. Mir N. U. Swalli J. S. Gee K. A. (2008). Is facial emotion recognition impairment in schizophrenia identical for different emotions? A signal detection analysis. *Schizophr. Res.* 99 263–269. 10.1016/j.schres.2007.11.006 18180142

[B49] Verbaan D. Marinus J. Visser M. Van Rooden S. M. Stiggelbout A. M. Middelkoop H. A. (2007). Cognitive impairment in Parkinson’s disease. *J. Neurol. Neurosurg. Psychiatry* 78 1182–1187. 10.1136/jnnp.2006.112367 17442759 PMC2117586

